# A Photocleavable Contrast Agent for Light-Responsive MRI

**DOI:** 10.3390/ph13100296

**Published:** 2020-10-08

**Authors:** Friederike Reeßing, Sèvrin E. M. Huijsse, Rudi A. J. O. Dierckx, Ben L. Feringa, Ronald J.H. Borra, Wiktor Szymański

**Affiliations:** 1 Department of Radiology, Medical Imaging Center, University Medical Center Groningen, University of Groningen, Hanzeplein 1, 9713GZ Groningen, The Netherlands; friederike.reessing@googlemail.com (F.R.); s.e.m.huijsse@umcg.nl (S.E.M.H.); r.a.dierckx@umcg.nl (R.A.J.O.D.); b.l.feringa@rug.nl (B.L.F.); r.j.h.borra@umcg.nl (R.J.H.B.); 2 Stratingh Institute for Chemistry, University of Groningen, Nijenborgh 4, 9747 AG Groningen, The Netherlands; 3 Department of Nuclear Medicine and Molecular Imaging, Medical Imaging Center, University Medical Center Groningen, University of Groningen, Hanzeplein 1, 9713GZ Groningen, The Netherlands

**Keywords:** magnetic resonance imaging, responsive MRI contrast agents, photoremovable protecting groups, relaxivity, light activation

## Abstract

Thanks to its innocuousness and high spatiotemporal resolution, light is used in several established and emerging applications in biomedicine. Among them is the modulation of magnetic resonance imaging (MRI) contrast agents’ relaxivity with the aim to increase the sensitivity, selectivity and amount of functional information obtained from this outstanding whole-body medical imaging technique. This approach requires the development of molecular contrast agents that show high relaxivity and strongly pronounced photo-responsiveness. To this end, we report here the design and synthesis of a light-activated MRI contrast agent, together with its evaluation using UV–vis spectroscopy, Fast Field Cycling (FFC) relaxometry and relaxometric measurements on clinical MRI scanners. The high relaxivity of the reported agent changes substantially upon irradiation with light, showing a 17% decrease in relaxivity at 0.23T upon irradiation with λ = 400 nm (violet) light for 60 min. On clinical MRI scanners (1.5T and 3.0T), irradiation leads to a decrease in relaxivity of 9% and 19% after 3 and 60 min, respectively. The molecular design presents an important blueprint for the development of light-activatable MRI contrast agents.

## 1. Introduction

Light is a powerful tool for the investigation of and control over biological functions. Due to its biocompatibility and the possibility of delivering it with very high spatiotemporal resolution, it has found many applications in modern medicine and biomedical research [1]. Well established and clinically implemented light-based modalities include the treatment of neonatal hyperbilirubinemia and the use of photodynamic therapy (PDT) in oncology and dermatology [2,3,4]. Furthermore, scientific literature comprises many more exploratory approaches to control biological systems or drug activity with light, such as photopharmacology [5,6] or optogenetics [7], both showing very promising results in pre-clinical studies.

Besides photo-controlled therapy, where irradiation is used to control the bioactivity of molecules [8], light also plays an essential role in clinical diagnostics: it is used as a readout signal or excitation stimulus in optical and optoacoustic imaging methods [9,10]. The most prominent optical imaging technique is fluorescence imaging, which relies on the detection of light emitted from fluorescent tracers after their excitation through irradiation at an appropriate wavelength [11,12,13,14,15]. Similarly, in (bio-)luminescence imaging, light from emitting moieties is recorded as a signal, but without the need to excite the respective tracer [16,17,18]. As light is inherently absent in the human body, luminescence imaging is characterized by outstanding sensitivity and specificity, as only the externally applied imaging agents generate a signal. Such agents are for example luciferin/luciferase- or horseradish peroxidase-based systems, which have been engineered for potential in vivo use in humans [19,20].

Despite the outstanding advantages of optical imaging, it also faces certain challenges: light is substantially absorbed and scattered in the human body, limiting the possible imaging depth and resolution of this imaging modality [1]. Conversely, alternative techniques, such as magnetic resonance imaging (MRI), allow whole body imaging with remarkably high resolution, albeit with much lower sensitivity [21]. Therefore, combining these two imaging modalities on a molecular level may enable high-resolution whole-body imaging with superior sensitivity. To this end, we describe here a photoresponsive MRI contrast agent (CA) that changes its relaxivity in response to external light irradiation. This simple molecular imaging agent has thus the potential to translate the signal generated, e.g., by optical/luminescence imaging agents to a readout suitable for MR imaging. An additional advantage of this approach is that one light-emitting moiety, which emits multiple photons, can activate multiple CA molecules, leading to signal amplification.

Several light-responsive MRI CAs have been reported in the last decade. For instance, Herges and co-workers reported porphyrin-based nickel(II) complexes, bearing a photoresponsive azobenzene moiety, whose relaxivity can be switched on by irradiation with λ = 505 nm light [22,23]. Even though the authors were able to minimize initial challenges with respect to the low water solubility, the overall very low relaxivity (0.03 s^−1^ mM^−1^ at 7T) remains a challenge [24]. Another light-responsive agent was developed by Louie and co-workers, and it employs a photoswitchable, spiropyrane-modified gadolinium complex [25]. This report successfully demonstrates the possibility of using bioluminescent tools (i.e., a luciferin/luciferase system) in order to provoke a photochemical transformation, in this case a cyclization reaction, lending credibility to the general approach. However, also in this case the overall relaxivity (≤ 2.93 s^−1^ mM^−1^ at ca. 1.4T) and more importantly, the change in relaxivity upon activation (ca. 10%) are relatively low and require further optimization.

**Figure 1:**
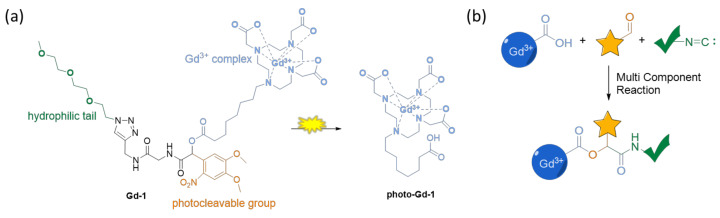
Design and synthetic approach to light-responsive MRI CA **Gd-1**. (**a**) Molecular structure of **Gd-1** and its photocleavage product; and (**b**) a general strategy for the synthesis of photoactivatable, gadolinium(III)-based CAs.

In this context, previous research performed in our group resulted in a liposomal, light-responsive MRI CA that gave very promising *in vitro* results not only for diagnostic applications but also for image-guided drug delivery [26]. Still, the general disadvantages of such nanoscopic systems are their high complexity and often suboptimal pharmacokinetic features, as their uptake into target tissues/cells might be limited. In order to tackle the problems of this design, we focused here on a development of a small molecule, exhibiting similar characteristics in terms of relaxivity to the liposomal system, but with a well-defined and straightforward structure. We synthesized the target molecule **Gd-1** (Figure 1) employing a Passerini multi-component reaction in the key step for the assembly of the photoactive core [27]. Thorough photochemical and relaxometric analysis revealed that irradiation with λ = 400 nm leads to photocleavage and a substantial change in relaxivity.

## 2. Results

Figure 1 illustrates the molecular design of photoresponsive MRI CA **Gd-1** that employs a nitro-veratryloxycarbonyl (NVOC)-based photocleavable core structure (Figure 1, highlighted in orange). This photocleavable protecting group (PPG) and its derivatives are amongst the most applied photoresponsive moieties and their properties have been thoroughly researched [28,29]. The application of NVOC-protected structures has enabled photo-control over protein function [30], dimerization [31] and degradation [32], as well as enzyme activity [33,34,35] and cytotoxicity [36].

Considering this extensive knowledge on the photochemical behavior and applications, we assumed that the irradiation of **Gd-1** with the light of an appropriate wavelength will cause the photocleavage of the NVOC-group, releasing the gadolinium(III) complex with a C_8_-alkyl chain featuring a terminal carboxylic acid (Figure 1a, blue). This carboxylic acid might coordinate to the gadolinium(III) center and replace one of the hydrating water molecules, causing a change in relaxivity [37,38,39,40]. Moreover, **Gd-1** comprises a hydrophilic triethyleneglycol chain (Figure 1a, green), which dictates the overall hydrophilicity of the intact compound, and is removed from the complex upon irradiation, which would result in the increased hydrophobicity of the released complex. Since it is known that not only the number of water molecules that directly coordinate to the metal center, but also the number of water molecules in the second and outer spheres that determine the relaxivity of a CA, we expected a significant change in relaxivity upon irradiation due to both factors [41,42,43].

**Figure 2:**
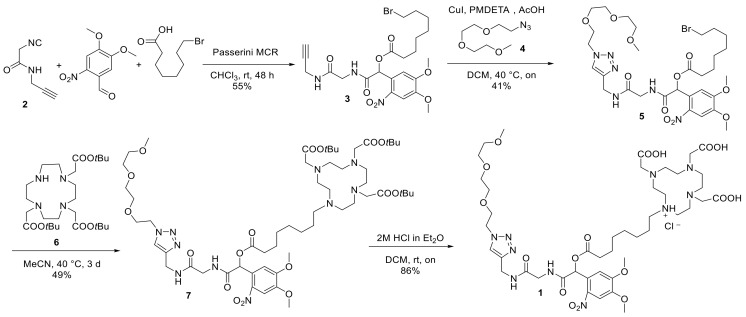
Synthetic route towards compound **1**.

The first step in the synthesis of compound **1** was the preparation of alkyne **2** with a pending isocyanide group, as reported previously [44]. Isocyanide **2** was then reacted in a Passerini multicomponent reaction with 2-nitroveratryl aldehyde and 8-bromooctanoic acid, yielding compound **3**, which comprises the photoactive core of the molecule (Figure 2). Subsequently, we connected the triethyleneglycol, featuring an azide functionality, to compound **3** in a copper(I)-catalyzed azide–alkyne cycloaddition (CuAAC). Of note, it was necessary to adjust the standard conditions of the CuAAC reaction, which normally include the use of a copper(II) salt with a reducing agent in aqueous medium, to assure the solubility of the starting materials and circumvent copper complexation by triethyleneglycol. Accordingly, the reaction was carried out in dichloromethane (DCM) with copper(I) iodide as a copper source and PMDTA (*N,N,N′,N″,N″-*pentamethyldiethylene-triamine) as its ligand [45], affording compound **5** in 69% yield. This product was then reacted with the previously synthesized gadolinium(III) ligand **6** in a nucleophilic substitution reaction [46]. Finally, deprotection of the *tert-*butyl groups afforded target compound **1** as the hydrochloride salt.

### 2.1. Photochemical Analysis

Since the photoresponsiveness of the synthesized agent is key for the performance of the designed photoactive MRI CA, we proceeded with the photochemical analysis of compound **7**. Based on the assumption that the *tert*-butyl-protecting groups on the 1,4,7,10-Tetraazacyclododecane-1,4,7,10-tetraacetate (DOTA) ligand do not significantly influence its photochemical behavior, we expected compounds **7** and **1** to have very similar photochemical characteristics, allowing the translation of the findings for more easily accessible compound **7** to its deprotected analogue. 

**Figure 3:**
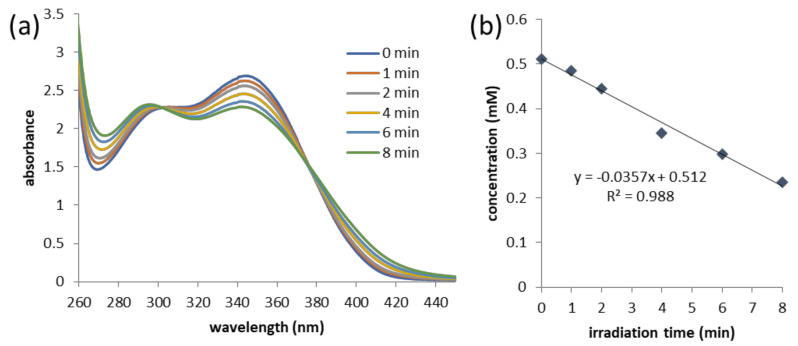
Photocleavage process and its quantum yield determination for compound **7** (0.565 mM in acetonitrile). (**a**) UV–vis spectra of compound **7** before (0 min) and upon irradiation with λ = 365 nm light for the indicated times (0.565 mM in acetonitrile); and (**b**) the remaining concentration of compound **7** after irradiation for indicated times.

The UV–vis absorption spectrum of compound **7** (Figure 3) features an absorption band of the NVOC core with a maximum at λ = 345 nm. Irradiation is expected to cause the photolysis of this group, which is made apparent by a decrease in absorbance at this wavelength. The efficiency of the photocleavage process is the product of the absorption coefficient, which is a measure for the probability that the molecule absorbs the light of the wavelength of interest (i.e., irradiation wavelength), and the photocleavage quantum yield (ϕ), which describes the chance that the absorbed energy causes photocleavage and will not be released in another way (e.g., via radiation or thermal relaxation). For the determination of the photocleavage quantum yield, a solution of compound **7** in acetonitrile (0.565 mM) was irradiated in a quartz cuvette with the light of λ = 365 nm for up to 8 min. Since the partial hydrolysis of the ester had been observed upon storage, especially of the deprotected compound **1**, the experiment was performed in pure acetonitrile assuring that the observed cleavage solely stems from photolysis. The process was monitored by UV–vis spectrometry and the photocleavage rate determined by UPLC (see Supporting Figure S1). Based on this analysis, the photocleavage quantum yield was calculated as 4.4%, indicating that the photocleavage efficiency of our photoactive scaffold lies in the same range as the one of comparable examples of NVOC-photocaged carboxylic acids, including methyl-substituted ones [47,48,49]. Still, it has to be noted that the quantum yield in aqueous medium possibly differs from the one in aprotic organic solvent, since—at least for photocaged alcohols—the cleavage mechanism is based on proton transfer and is known to be affected by changes in pH and solvent.

### 2.2. Fast Field Cycling Relaxometric Analysis

**Figure 4:**
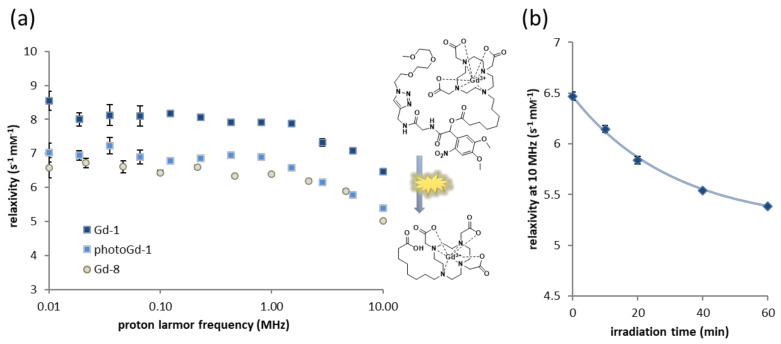
Relaxometric analysis of **Gd-1**, **photo-Gd-1** and **Gd-8** in TBS buffer (0.8 mM). (**a**) NMRD profiles of **Gd-1** before irradiation (dark blue) and after exposure to λ = 400 nm light for 60 min (light blue) and **Gd-8** (gray); (**b**) decrease in the relaxivity of a sample of **Gd-1** at 10 MHz in response to irradiation for the indicated times. The line represents an exponential fit of the data. The error bars represent the uncertainty of fitting the T_1_ curve to the experimental data.

With the assumption that compound **1** undergoes efficient photocleavage, in a manner analogous to the model compound **7**, we proceeded to assess its light-dependent relaxivity. For this purpose, we formed the gadolinium(III) complex of compound **1** (**Gd-1**) in TBS buffer at pH 7.5. In order to assure the full complexation of gadolinium(III), an excess of the ligand (1.8 eq.) was used and the absence of free gadolinium(III) verified as described in Section 2.4. With the gadolinium(III) complex in hand, we used FFC relaxometry to evaluate the performance of the agent in terms of relaxation enhancement. The respective Nuclear Magnetic Relaxation Dispersion (NMRD) profile of **Gd-1** is depicted in Figure 4 (see Supporting Table S1 for numerical data), revealing the higher relaxivity of our probe as compared to other light-responsive MRI CAs described earlier.

The effect of irradiation on the relaxivity is the crucial feature of the developed agent. Therefore, we irradiated the sample with λ = 400 nm light for 60 min in total and monitored the NMRD profiles. As illustrated in Figure 4, irradiation leads to a clear decline in relaxivity over the whole spectrum of the recorded (proton) Larmor frequencies (0.01–10 MHz). At 10 MHz (Figure 4b), the total decrease in relaxivity from 6.47 s^−1^ mM^−1^ to 5.39 s^−1^ mM^−1^ constitutes a 17% change. Importantly, we also analyzed the relaxometric properties of the expected photocleavage product (**Gd-8**) for comparison with the actual photoproduct (**photo-Gd-1**). Ligand **8** had been synthesized previously and the complex prepared following the same procedure as for **Gd-1**. Figure 4 shows the respective NMRD profile depicted in gray (see Supporting Table S2 for numerical data). Evidently, the profile of the irradiated sample converges to a large extent to the profile of the model compound, suggesting that **Gd-8** is indeed the photocleavage product. Kinetic analysis of the decrease in relaxivity at 10 MHz shows an exponential decay with a calculated lifetime of 31 min (half-life: 21.5 min).

### 2.3. Relaxometric Measurements on Clinical 1.5T and 3.0T Systems

**Figure 5:**
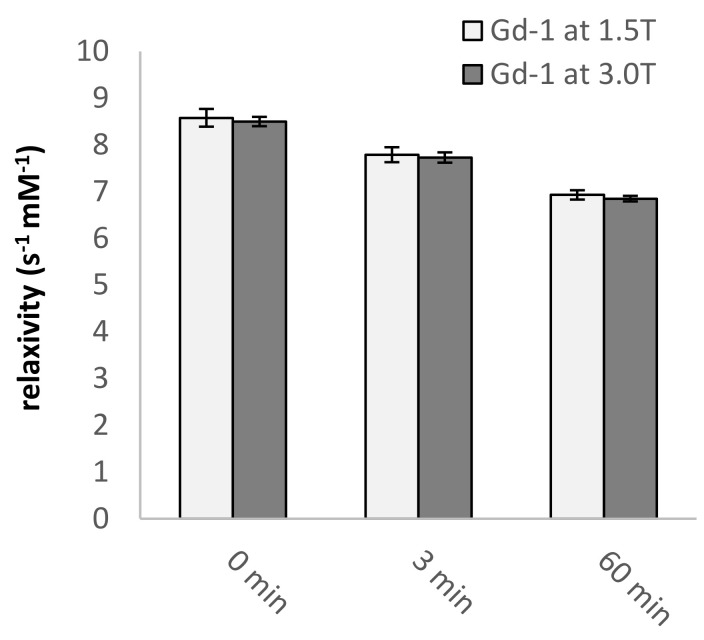
The relaxivity of a sample of **Gd-1** (0.35 mM) in TBS buffer in response to irradiation at λ = 365 nm light for the indicated times, at 1.5T and 3.0T. Bars represent the mean ± standard deviation.

As described in Section 2.3, the irradiation of the developed CA leads to a clear decline in relaxivity in the range of 0.01–10 MHz. To determine if the same effect can be observed for clinically applied magnetic field strengths, the irradiated compounds were measured on 1.5T (63.87 MHz) and 3.0T (127.74 MHz) MRI scanners. Here, we additionally evaluated the use of lower power irradiation source (3 mW Fiber-coupled LED, λ = 365 nm), to better represent the lower photon fluxes that can be achieved in a biological context [1]. As illustrated in Figure 5**,** the irradiation of **Gd-1** leads to a clear decrease in relaxivity—and therefore an increase in T_1_ relaxation time—at both magnetic field strengths, with a decrease in relaxivity of 9% and 19% upon irradiation for 3 and 60 min, respectively. Furthermore, **Gd-1** shows the high molar relaxivity of >8 s^−1^ mM^−1^, superior to most of the clinically used contrast agents, which feature relaxivities of 3–6 s^−1^ mM^−1^ [50].

### 2.4. Assessment of Free Gadolinium(III) Ions

Since there is an increasing concern about the liberation of free gadolinium(III) from MRI CAs and its accumulation in the body, we tested if there is any free gadolinium(III) present in the sample before irradiation and if irradiation leads to a release of free gadolinium(III) from the complex. Towards this end, we employed a photometric assay based on xylenol orange [51]. The experiment confirmed the absence of free gadolinium(III) ions in an irradiated sample of **Gd-1**. This finding assured the validity of our ligand design, providing a base for the further development of responsive gadolinium-based CAs.

## 3. Discussion

We designed, synthesized, and evaluated a photoresponsive MRI CA that shows a 17% decrease in relaxivity at 0.23T (10 MHz) upon irradiation with λ = 400 nm (violet) light for 60 min. On clinical MRI scanners with magnetic field strengths of 1.5T and 3.0T, the CA leads to a decrease in relaxivity of 9% and 19% after 3 and 60 min of irradiation, respectively, when using an inversion recovery MRI sequence for the relaxometric measurements. This effect probably stems from a change in the number of water molecules hydrating the gadolinium(III) complex in the outer sphere, as well as in the first and second sphere. Moreover, the reduced molecular weight of the photocleavage product, with respect to the initial molecule, may play a role in the reduced relaxivity and thus increased longitudinal relaxation time. As compared to the few previously described photoresponsive MRI CAs, the presented design is characterized by high relaxivity and relatively well pronounced change in its magnetic properties under irradiation.

The research presented herein essentially substantiates the proof of principle for small molecule light-activated MRI contrast enhancement and adds valuable analytical insights into the photo-induced modulation of relaxivity. In the future, the design may set the base for agents with improved features, especially in terms of activation wavelength, uncaging efficiency and reversing the contrast agent to a “switch on” instead of “switch off” mode. Towards this end, the replacement of the NVOC-group by another photocleavable group, like a boron-dipyrromethene (BODIPY)-based one, would render the CA responsive to red or NIR-light, minimizing the harmful effects of the employed irradiation on the surrounding tissue [52,53]. A “switch on” MRI CA could be established by designing a molecule that bears a coordinating moiety, such as a carboxylic acid, which will be cleaved off upon irradiation. In this manner, an increase in hydration number and thus an increase in relaxivity could be generated. We have also shown that the irradiation of the CA results in a change of relaxation time that can be detected by clinical MRI scanners. However, the inversion recovery sequence, which was used for the quantitative relaxometric measurements, was very time consuming and is therefore not clinically applicable. In the future, MRI sequences may be explored that allow for the quantitative measurement of relaxation times, with a scan time that is clinically applicable, or conventional non-quantitative clinically used T_1_-weighted MRI sequences might be applied.

## 4. Materials and Methods

### 4.1. General Information

Starting materials, reagents and solvents were purchased from Sigma–Aldrich, Acros, Fluka, Fisher Scientific, TCI and were used as received. Solvents for the reactions were of p.a. purity. Anhydrous solvents were purified by passage through solvent purification columns (MBraun SPS-800, Garching, Germany). For aqueous solutions, deionized water was used. Thin layer chromatography (TLC) analyses were performed on commercial Kieselgel 60, F_254_ silica gel plates with fluorescence-indicator UV254 (Merck, TLC silica gel 60 F_254_, Darmstadt, Germany). For the detection of components, UV light at λ = 254 nm or λ = 365 nm was used. Alternatively, oxidative staining using aqueous basic potassium permanganate solution (KMnO_4_) or aqueous acidic cerium phosphomolybdic acid solution (Seebach’s stain) was used. The drying of solutions was performed with MgSO_4_ and volatiles were removed with a rotary evaporator. Flash column chromatography was performed with silica gel, pore size 60 Å, 40–63 µm in particle size.

Nuclear magnetic resonance spectra were measured with an Agilent Technologies 400-MR (Santa Clara, CA, USA) (400/54 Premium Shielded) spectrometer (400 MHz). All spectra were measured at room temperature (22–24 °C). The multiplicities of the signals are denoted by s (singlet), d (doublet), t (triplet), q (quartet), quint (quintet), m (multiplet), br (broad signal). All ^13^C-NMR spectra are ^1^H-broadband decoupled. High-resolution mass spectrometric measurements were performed using a Thermo scientific LTQ OrbitrapXL spectrometer with ESI ionization. The ions are given in m/z-units. Melting points were recorded using a Stuart analogue capillary melting point SMP11 apparatus. For the spectroscopic measurements, solutions in Uvasol® grade solvents were measured in a 10 mm quartz cuvette. UV–vis absorption spectra were recorded on a JascoV-750 UV–vis spectrophotometer with photomultiplier tube detection. UV–vis absorbance of the photometric assay for Gd^III^ quantification were performed on a BioTek Synergy H1 microplate reader (Winooski, VT, USA). NMRD profiles were recorded on a Stelar 0.25T FFC SMARtracer relaxometer (Mede, Italy). UPLC–MS analysis was performed using a ThermoFisher Scientific Vanquish UPLC System (Waltham, MA, USA) with a reversed phase C18 column (Acquity UPLC HSS T3 1.8 μm, 2.1 × 150 mm; eluents: water and acetonitrile, both with 0.1% *v*/*v* formic acid added; the gradient was established from 5% to 95% organic phase over 17 min) in combination with an LCQ Fleet mass spectrometer and UV–vis detector at 360 nm.

Irradiation experiments were performed with a λ = 400 nm LED system (3× Roithner VL-400-Emitter, optical power 1000 mW, λ_max_ = 400 nm, Full Width at Half Maximum (FWHM) 11.9 nm, Sahlmann Photochemical Solutions, Bad Segeberg, Germany) and a λ = 365 nm ThorLabs M365F1 3.0 mW fiber-coupled LED (Newton, NJ, USA).

Quantum yield determination was performed using a custom-built (Prizmatix/Mountain Photonics, Landsberg am Lech, Germany) multi-wavelength fiber-coupled LED system (FC6-LED-WL) using 365A LED. The FWHM was ≤ 20 nm. The LED was connected through a 7 to 1 fiber bundle attached to a 3 mm liquid light guide (LLG-3) and a liquid light guide adapter (LLG-AC). The adapter was placed in a Thorlabs SMR1 lens mount, which was adjusted to height using Thorlabs TR20/30 optical posts, AS6M4M adapters and a PJ302/M Offset Mounting Post Joist. For all kinetic experiments, the temperature was maintained at 293K using a Quantum Northwest TC1 temperature controller.

### 4.2. Synthetic Procedures and Spectroscopic Data

For 1-(4,5-Dimethoxy-2-nitrophenyl)-2-oxo-2-((2-oxo-2-(prop-2-yn-1-ylamino)ethyl)amino)-ethyl 8-bromooctanoate (3), a solution of **2** [44] (4.06 mmol, 500 mg), 6-nitroveratraldehyde (3.37 mmol, 714 mg) and 8-bromooctanoic acid (4.06 mmol, 905 mg) in chloroform (8 mL) was stirred at room temperature for 48 h. The volatiles were evaporated, and the product was purified by flash chromatography (pentane/AcOEt, 95:5 to 1:1, *v*/*v*) to give a yellow powder (1038 mg, 55%). R*_f_* = 0.80 (AcOEt); Mp. 106–107 °C; ^1^H NMR (400 MHz, DMSO): *δ* 1.26–1.35 (m, 6H), 1.53 (m, 2H), 1.76 (m, 2H), 2.41 (t, 2H), 3.12 (s, 1H), 3.51 (t, 2H), 3.76 (d, 2H), 3.87 (d, 2H), 3.88 (s, 3H), 3.91 (s, 3H), 6.59 (s, 1H), 7.15 (s, 1H), 7.65 (s, 1H,), 8.39 (t, 1H), 8.59 (t, 1H); ^13^C NMR (100 MHz, CDCl_3_): *δ* 24.6, 27.9, 28.4, 28.8, 29.3, 32.6, 33.8, 33.9, 43.2, 56.5, 56.7, 71.0, 71.6, 78.6, 107.9, 111.2, 124.4, 140.5, 149.2, 153.7, 167.9, 168.1, 173.0; HRMS (ESI-) calc. for [M]^−^ (C_23_H_31_BrN_3_O_8_) : 556.1289, found: 556, 1275.

For 1-(4,5-Dimethoxy-2-nitrophenyl)-2-((2-(((1-(2-(2-(2-methoxyethoxy)ethoxy)ethyl)-1*H*-1,2,3-triazol-4-yl)methyl)amino)-2-oxoethyl)amino)-2-oxoethyl 8-bromooctanoate (5), to a solution of **3** (0.44 mmol, 249 mg), and **4** (0.74 mmol, 140 mg) were added PMDETA (0.04 mmol, 9.2 µL), catalytic amounts of copper iodide and ascorbic acid and a drop of acetic acid. The reaction mixture was stirred at room temperature for three days. The conversion of **3** was monitored by TLC. After two days, another portion of **4**, copper iodide, acetic acid and ascorbic acid were added. After the full conversion of **3** was determined (TLC), the DCM and H_2_O were added to the reaction mixture. The product was extracted with DCM (3x). The combined organic layers were washed with H_2_O and brine and the product was purified by flash column chromatography (DCM/MeOH, 98:2–93:7, *v*/*v*) to obtain the product as a yellow sticky solid (134 mg, 41%). R*_f_* = 0.68 (DCM/MeOH, 9:1, *v*/*v*); ^1^H NMR (400 MHz, CHCl_3_): *δ* 1.30–1.40 (m, 6H), 1.63 (m, 2H), 1.82 (m, 2H), 2.43 (m, 2H), 3.36 (s, 3H), 3.38 (t, 2H), 3.54 (m, 2H), 3.60 (m, 6H), 3.86 (m, 2H), 3.91–4.07 (m, 1H), 3.94, (s, 3H), 3.98 (s, 3H), 4.50 (m, 4H), 6.68 (s, 1H), 7.04 (t, 1H), 7.16 (s, 1H), 7.28 (t, 1H), 7.58 (s, 1H), 7.71 (s, 1H); ^13^C NMR (100 MHz, CDCl_3_): *δ* 24.7, 28.0, 28.4, 28.9, 32.7, 34.0, 34.0, 35.2, 43.1, 50.4, 56.6, 56.8, 59.1, 69.5, 70.6 (m), 70.6, 71.0, 72.0, 108.1, 111.3, 123.5, 124.9, 140.8, 144.2, 149.2, 153.7, 167.9, 168.3, 172.7; HRMS (ESI+) calc. for [M+H]^+^ (C_30_H_46_BrN_6_O_11_): 745.2403, found: 745.2406.

For Tri-*tert*-butyl 2,2′,2″-(10-(8-(1-(4,5-dimethoxy-2-nitrophenyl)-2-((2-(((1-(2-(2-(2-methoxy ethoxy)ethoxy)ethyl)-1*H*-1,2,3-triazol-4-yl)methyl)amino)-2-oxoethyl)amino)-2-oxoethoxy)-8-oxo octyl)-1,4,7,10-tetraazacyclododecane-1,4,7-triyl)triacetate (7), compound **6** (0.42 mmol, 250 mg) was suspended in H_2_O at 70 °C. The heating bath was removed and 10% aq. Potassium hydroxide (0.84 mmol, 0.47 mL) was added. The mixture was stirred for 15 min and then extracted with pentane (3x). The combined organic layers were washed with H_2_O (2×) and brine (1×) and dried with MgSO_4_. The solvent was evaporated and the residue (0.24 mmol, 124 mg) dissolved in acetonitrile. Compound **5** (0.19 mmol, 140 mg) was added and the solution was stirred at 40 °C for three days. Afterwards, the solvent was evaporated, and the product purified by flash column chromatography (DCM/MeOH, 10:0–9:1, *v*/*v*) to give a yellow sticky oil (109 mg, 49%). R*_f_* = 0.62 (DCM/MeOH, 9:1, *v*/*v*); ^1^H NMR (400 MHz, CHCl_3_): *δ* 1.23–1.36 (m, 9H), 1.41–1.45 (m, 27H), 1.58 (m, 2H), 2.16–3.14 (m, 23H), 2.39–2.64 (m, 2H), 3.35 (s, 3H), 3.51–3.52 (m, 2H), 3.57–3.59 (m, 6H), 3.84 (t, 2H), 3.91 (s, 3H)*, 3.96 (s, 3H)*, 4.00–4.07 (m, 2H), 4.48 (t, 2H), 4.52 (t, 2H), 6.78 (s, 1H)*, 7.2 (s, 1H)*, 7.46 (t, 1H), 7.55 (s, 1H)*, 7.83 (s, 1H)*, 7.92 (t, 1H), *the assigned signals split up, probably because of the existence of two diastereoisomers due to the atropoisomerism that stems from the hindered rotation of the *ortho*-nitro phenyl group; ^13^C NMR (100 MHz, CDCl_3_): *δ* 24.5, 26.4, 27.3, 27.9, 28.0, 28.1, 28.2, 28.3, 28.9, 29.2, 33.8, 34.1, 35.4, 43.2, 47.9, 50.2, 50.4, 53.3, 54.4, 55.8, 56.5, 57.0, 59.1, 69.5, 69.5, 70.5, 70.6, 70.7, 71.0, 72.0, 77.2, 81.8, 81.9, 82.6, 83.0, 108.1, 108.2, 111.4, 123.5, 123.6, 125.3, 141.2, 145.1, 149.0, 153.5, 168.0, 168.9, 169.0, 170.1, 170.6, 172.6, 172.8, 173.0; HRMS (ESI+) calc. for [M+H]^+^ (C_56_H_95_N_10_O_17_): 1179.6871, found: 1179.6900.

For 2,2′,2″-(10-(8-(1-(4,5-Dimethoxy-2-nitrophenyl)-2-((2-(((1-(2-(2-(2-methoxy-ethoxy)ethoxy) ethyl)-1*H*-1,2,3-triazol-4-yl)methyl)amino)-2-oxoethyl)amino)-2-oxo-ethoxy)-8-oxooctyl)-1,4,7,10-tetraazacyclododecane-1,4,7-triyl)triacetic acid (1), a solution of **7** (0.013 mmol, 15 mg) in DCM (0.5 mL), HCl in Et_2_O (2 M, 0.5 mL) and tri-*iso*-propylsilane (0.02 mL) was stirred at room temperature overnight. The volatiles were evaporated under reduced pressure. The residue was triturated with Et_2_O and washed with Et_2_O and pentane to give a yellow sticky solid (11.7 mg, 86% calculated as mono hydrochloride salt). The ^1^H NMR (400 MHz, MeOD): *δ* 1.37 (m, 6H), 1.63 (m, 2H), 1.82 (m, 2H), 2.49 (m, 2H), 2.95–3.06 (m, 4H), 3.13–3.26 (m, 6H), 3.34 (s, 3H), 3.38–3.52 (m, 9H), 3.63–3.55 (m, 12H), 3.88–3.94 (m, 4H), 3.93 (s, 3H), 3.96 (s, 3H), 4.25 (s, 2H), 4.52 (d, 2H), 4.62 (t, 2H), 6.79 (s, 1H), 7.26 (s, 1H), 7.69 (s, 1H), 8.11 (s, 1H). ^13^C NMR (100 MHz, MeOD) *δ* 24.4, 25.6, 27.3, 29.6, 29.6, 34.6, 35.1, 43.5, 49.6, 50.0, 51.2, 52.4, 53.1, 53.4, 55.6, 56.1, 57.3, 59.1, 70.0, 71.3, 71.4, 72.2, 72.9, 109.5, 113.0, 125.7, 126.2, 142.8, 150.7, 154.8, 168.6, 170.9, 171.1, 174.1, 174.8; HRMS (ESI+) calc. for [M+H]^+^: (C_44_H_71_N_10_O_17_): 1011.4993, found: 1011.4997.

Synthetic procedures and spectroscopic data for further intermediates can be found in the Supplementary Materials.

### 4.3. Quantum Yield Determination

The quantum yield of the photocleavage process of compound **7** was determined following the photo-deprotection process by UPLC–MS. A solution of compound **7** (565 µM) in acetonitrile (3.00 mL) was irradiated in a quartz cuvette with a multi-wavelength fiber-coupled LED system (FC6-LED-WL) using a 365A LED. The photon flux (I = 4.059 × 10^−8^ mol s^−1^) was determined previously by ferrioxalate actinometry following a modified literature protocol [54]. During the irradiation, the solution was vigorously stirred in order to ensure homogenous concentration. The temperature was kept constant at 25 °C. The high concentration of compound **7** enabled us to work in a high-absorption regime (absorbance at 365 nm ≥ 1.9) and assume that all incident photons were absorbed. Aliquots of 10 μL of the irradiated solution were taken at the indicated time points, diluted with acetonitrile (140 μL) and analyzed by UPLC–MS. The remaining concentration of compound **7** was quantified by the determination of the peak area of the corresponding peak in the chromatogram recorded at 365 nm using a calibration curve (see Figure S1). This way, the rate of photocleavage was found to be 6 × 10^−7^ M s^−1^ and the respective quantum yield was calculated using the following equation: the calculation of the photocleavage quantum yield; Δc = change in concentration (M s^−1^); V = sample volume (L):

$$\frac{\Delta c\times V}{I}=\Phi$$

$$\frac{6\times 10^{-7}\;M\;s^{-1}\times 0.003\;L}{4.059\times 10^{-8}\;\mathrm{mol}\;s^{-1}}\;=\;0.044,\;\mathrm{corresponding}\;\mathrm{to}\;4.4\%$$

### 4.4. FFC Relaxometry

The relaxation rates were determined over a (proton) Larmor frequency range of 0.01–10 MHz at 37 °C with 12 data points collected. The samples were prepared by mixing 1 eq. (1.44 mM) of compound **1** or compound **8**, with 0.56 eq. (0.8 mM) of GdCl_3_ in TBS buffer at room temperature for 2 h. The samples were irradiated in the NMRD vessel with λ = 400 nm light for 60 min. NMRD profiles of one aliquot per sample were recorded before irradiation and after 10, 20, 40 and 60 min of irradiation time points (see Supporting Figure S2 and Supporting Table S1, where the SD values correspond to the uncertainty of the T_1_ curve fitting). In addition, the stability of the sample was assessed by repeating the analysis after leaving the sample for 1 h at room temperature without irradiation.

### 4.5. Measurements on 1.5T and 3.0T Clinical MRI Systems

**Gd-1** was prepared by mixing 1.8 equivalents of compound **1** with one equivalent of GdCl_3_ in TBS buffer (pH 7.5, 5 mL). Complexation was assumed to be complete after 2 h, affording a molar concentration of **Gd-1** of 0.35 mM. The resulting solution was irradiated with λ = 365 nm light and aliquots of 1.6 mL were taken at the indicated time points for the determination of the relaxation rates. For the Gd-control sample, a solution of equimolar gadolinium(III) concentration of GdCl_3_ in TBS buffer (pH 7.5) was prepared.

The irradiated samples (0, 3 and 60 min irradiation time points) were scanned at 1.5T and 3.0T (AvantoFit and Prisma, Siemens Healthcare, Erlangen, Germany) by using the MultiSample 120 phantom (Gold Standard Phantoms, London, UK; https://www.goldstandardphantoms.com/multisample120) at room temperature (21–22 °C), holding 15 mL Falcon conical tubes with 1.5 mL Eppendorf tubes containing the samples submerged in demineralized water. The MultiSample 120 phantom was positioned vertically in a 20-channel head coil with the lid downwards. Both the Eppendorf tubes and the Falcon tubes were positioned with the conical tip upwards so that the Eppendorf tubes were floating upwards into the conical shaped tip of the Falcon tubes. This allowed for the maximum achievable fixation of the vertically positioned Eppendorf tubes. In addition to the irradiated samples an Eppendorf with GdCl_3_ solution was included.

T_1_ relaxation time was measured using a coronal slice positioned at the height of the Eppendorf tubes, using a fast spin-echo (2DFSE) inversion recovery (IR) sequence with repetition time (TR) 4000 ms, echo time (TE) 13 ms and inversion times (TI) 50, 75, 100, 125, 150, 250, 500, 1000, 1500, 2000 and 3000 ms. Total acquisition time was 3 h and 9 min. Data were acquired with a field of view (FOV) of 150 mm with matrix size 256x256 and a slice thickness of 8 mm.

The modulus/magnitude IR data were analyzed by using the T_1_ relaxation analysis module of nordicICE (v4.2.0; NordicNeuroLab, Bergen, Norway), while applying correction for an imperfect inversion pulse and a baseline offset. No noise reduction or noise level detection/cutoff was applied. The T_1_ maps as calculated by nordicICE were exported and then imported into ImageJ (1.48v; National Institutes of Health, Bethesda, MD, USA) to place spherically shaped regions-of-interest (ROIs) with an area of 17.85 mm^2^ in the Eppendorf tubes. Mean and standard deviation of the T_1_ relaxation times of each ROI were noted and converted to relaxivity (see Supporting Table S3) by calculating the reciprocal of the relaxation time (relaxation rate R_1_ = 1/T_1_) divided by the concentration of **Gd-1**.

### 4.6. Determination of Free Gadolinium(III) Concentration

The concentration of free Gd^III^ was quantified by the determination of the ratio of absorbance intensity at λ = 573 nm and λ = 433 nm of a Gd^III^-xylenol orange complex in ammonium acetate buffer (100 mM, pH 5.8, 0.60 mM Xylenol Orange, see supporting Figure S3) using a microplate reader (see Figure S3) [50]. In order to assess how many equivalents of ligand are needed for the full complexation of Gd^III^, increasing equivalents of GdCl_3_ were added to a 0.3 mM solution of compound **1** in ammonium acetate buffer and the amount of free Gd^III^ was analyzed after 2 h by the addition of xylenol orange.

For the quantification of free Gd^III^ after irradiation under the conditions used for the relaxometric analysis, the complex was prepared and treated as described above (irradiation in the NMRD vessel, λ = 400 nm, 60 min). For analysis, the sample was diluted 1:30 (*v*/*v*) with ammonium acetate buffer. The concentration of free Gd^III^ was determined three times in independent measurements and found to be 0.54 µM on average.

## 5. Conclusions

Here, we present the development of a small molecule MRI contrast agents that reacts to light and changes its relaxivity at magnetic fields relevant in clinical use. Light-responsive MRI CAs constitute the first step in establishing a methodology for improving MRI sensitivity through signal amplification [26]. Their envisioned use is based on a two-step application of the imaging agent(s): Firstly, a light-emitting targeting moiety, i.e., an antibody aimed at antigens that are overexpressed in the tissue of interest, such as endothelial growth factor receptors in tumor tissue, would be injected. As mentioned above, suitable luminescent agents for this purpose would be luciferin/luciferase- or horseradish peroxidase-based systems. In a second step, the MRI CA agent would be administered which is consequently only being activated in the areas targeted by the light-emitting system. After further improvement of the molecular structure of the CA in terms of relaxivity change and light-responsiveness, we aim to endow this type of CA with additional functionality by substantiating on our efforts to combine MR imaging with drug delivery [26].

## Supplementary Materials

The following are available online at https://www.mdpi.com/1424-8247/13/10/296/s1: Synthetic procedures and NMR spectroscopic data for all new compounds, Figure S1: Quantification of compound **7** by UPLC, Figure S2: NMRD profiles of a sample containing **Gd-1** before irradiation (0 min), after 1 h in the dark (stability 1 h) and after irradiation for the indicated time points (10; 20; 40; 60 min), Figure S3: Quantification of free Gd^III^, Table S1: Molar relaxivity (s^−1^ mM^−1^) profiles of a sample of **Gd-1** in TBS buffer pH 7.5 before irradiation (0 min), after 1 h in the dark (stability 1 h) and after irradiation for the indicated time points (10; 20; 40; 60 min), Table S2: Molar relaxivity (s^−1^ mM^−1^) profile of a sample of **Gd-8** in TBS buffer pH 7.5, Table S3: Molar relaxivity (s^−1^ mM^−1^) and standard deviation (SD) of a sample of **Gd-1** in TBS buffer pH 7.5 in response to irradiation for the indicated time points (0; 3; 60 min) and **GdCl_3_**, at 1.5T (63.87 MHz) and 3.0T (127.74 MHz).
