# Supplementary material for: A Photocleavable Contrast Agent for Light-Responsive MRI
Source: Pharmaceuticals (Basel). 2020 Oct 8;13(10):296. doi: 10.3390/ph13100296 (PMC7599822; doi:10.3390/ph13100296)
Supplement: Supplementary file 1 [file pharmaceuticals-13-00296-s001.pdf]

Supplementary Materials

A Photocleavable Contrast Agent for Light-responsive MRI

Friederike Reeßing [1,3], Sèvrin E. M. Huijsse [1], Rudi A. J. O. Dierckx [1], Ben L. Feringa [1,3], Ronald J.H. Borra [1,2] and Wiktor Szymański [1,3,*]

[1] Department of Radiology, Medical Imaging Center, University Medical Center Groningen, University of Groningen, Hanzeplein 1, 9713GZ Groningen, The Netherlands
[2] Department of Nuclear Medicine and Molecular Imaging, Medical Imaging Center, University Medical Center Groningen, University of Groningen, Hanzeplein 1, 9713GZ Groningen, The Netherlands
[3] Stratingh Institute for Chemistry, University of Groningen, Nijenborgh 4, 9747 AG Groningen, The Netherlands
* w.szymanski@umcg.nl

1. Synthetic procedures and spectroscopic data

2-Isocyano-*N*-(prop-2-yn-1-yl)acetamide (2): Compound **2** was prepared by a modification of a literature procedure [1,2]. A mixture of methyl isocyanoacetate (7.82 mmol, 711 μL) and propargylamine (15.6 mmol, 1.00 mL) was stirred at room temperature overnight. The formed solid was filtered off and washed with Et_2O to provide compound **2** as light brown powder (759 mg, 78%). R_f = 0.63 (pentane/AcOEt, 1:1, v/v); Mp. 109°C; ^1H NMR (400 MHz, DMSO-d_6): δ 3.15 (t, 1H), 3.88 (dd, 2H), 4.35 (s, 2H), 8.61 (br s, 1H). ^1H NMR spectrum is in agreement with published data [1].

1-Azido-2-(2-(2-methoxyethoxy)ethoxy)ethane (4): 1-Bromo-2-(2-(2-methoxyethoxy) ethoxy)ethane (4.4 mmol, 1000 mg) was dissolved in ethanol (80 mL) and NaN_3 (8.8 mmol, 584 mg) was added. After heating the solution under reflux overnight, the solvent was evaporated and DCM added to the residue. The organic layer was washed with H_2O (3x) and dried with $MgSO_4$ to give a colorless liquid (758 mg, 91%). R_f = 0.67 (Pentane/AcOEt 1:1); ^1H NMR (400 MHz, $CHCl_3$): δ 3.62-3.58 (m, 8H), 3.48 (t, 2H), 3.33-3.31 (m, 5H). ^1H NMR spectrum is in agreement with published data [3]. ^{13}C NMR (100 MHz, $CDCl_3$): δ 71.9, 70.7, 70.6, 70.6, 70.0, 59.0, 50.7.

4,7,10-Tris(2-(*tert*-butoxy)-2-oxoethyl)-1,4,7,10-tetraazacyclododecan-1-ium bromide (6): The compound was prepared according to a literature procedure [4]. A suspension of cyclen (5.81 mmol, 1.00 g) and sodium acetate (19.1 mmol, 1.57 g) in *N,N*-dimethylacetamide (DMA, 12 mL) was stirred at −20 °C. A solution of *tert*-butyl bromoacetate (19.2 mmol, 2.83 mL) in DMA (4 mL) was added dropwise over 15 min at -20 °C. The reaction mixture was stirred at room temperature for 24 h, after which it was poured into H_2O (60 mL), resulting in a clear solution. Solid $KHCO_3$ (30.0 mmol, 3.00 g) was added portion-wise and a precipitate was formed. The precipitate was collected by filtration and dissolved in DCM (5 mL) and the solution was washed with H_2O (20 mL), dried over $MgSO_4$, filtered and concentrated to about 4-5 mL. Diethyl ether (50 mL) was added and compound **6** precipitated as a white solid (1.64 g, 48%). R_f = 0.63 (DCM/MeOH, 9:1, v/v); Mp. 179-181 °C; ^1H NMR (500 MHz, $CDCl_3$) δ 1.45 (s, 9H), 1.46 (s, 18H), 2.87 (m, 4H), 2.93 (m, 8H), 3.10 (m, 4H), 3.29 (s, 2H), 3.38 (s, 4H), 10.03 (br s, 1H). ^1H NMR spectrum is in agreement with published data [4].

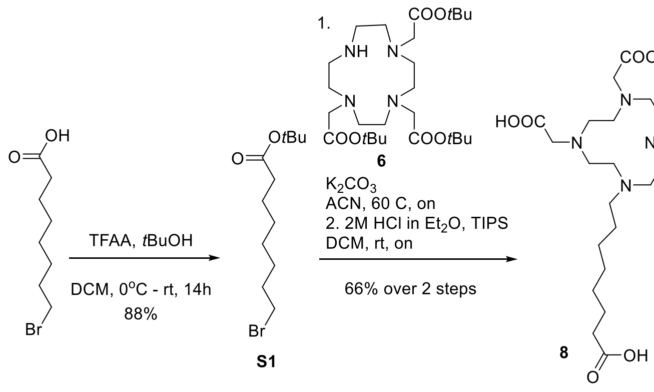

***tert*-Butyl 8-bromooctanoate (S1):** The compound was prepared according to a literature procedure [5]. 8-Bromooctanoic acid (1.57 mmol, 350 mg) was dissolved in dry DCM (15 mL) under nitrogen atmosphere. The resulting solution was cooled down to 0 ^{0}C and trifluoroacetic anhydride (3.61 mmol, 0.51 mL) was added dropwise. The reaction mixture was stirred at 0 ^{0}C for 2.5 h after which *tert*-butanol (5.5 mmol, 0.52 mL) was added slowly. The mixture was stirred at 0 ^{0}C for 1 h and was then allowed to warm to room temperature. The reaction mixture was stirred for additional 12 h at room temperature and then quenched with H_2O (10 mL). The product was extracted with Et_2O (3x) and the combined organic layers washed with brine, sat. aq. $NaHCO_3$ solution and dried with $MgSO_4$. The volatiles were evaporated to obtain compound **8** as a clear oil (384 mg, 88%). R_f = 0.9 (pentane/Et_2O, 4:1, v/v), ^{1}H NMR (400 MHz, $CDCl_3$): δ 1.31-1.34 (m, 6H), 1.44 (s, 9H), 1.55-1.62 (m, 2H), 1.85 (quint, 2H), 2.20 (t, 2H), 3.40 (t, 2H). ^{1}H NMR spectrum is in agreement with published data [5].

Tri-*tert*-butyl 2,2',2''-(10-(8-(*tert*-butoxy)-8-oxooctyl)-1,4,7,10-tetraazacyclo dodecane-1,4,7-triyl)triacetate (S2): Compounds **S1** (0.54 mmol, 150 mg) and **6** (0.27 mmol, 160 mg) were dissolved in acetonitrile and K_sCO_3 (1.1 mg, 148 mg) was added to the solution. The reaction mixture was stirred at 60 ^{0}C overnight. The salts were filtered off, the volatiles evaporated and the product was purified by flash column chromatography (DCM/MeOH 98:2 – 9:1 v/v) to give compound **S1** (131 mg, 68%). R_f = 0.6 (DCM/MeOH, 9:1, v/v); HRMS (ESI+) calc. for $[M+H]^+$ ($C_{38}H_{72}N_4O_8$): 713.5423, found: 713.5431; ^{1}H NMR (400 MHz, $CDCl_3$): δ 1.31-3.41 (overlapping br m), 1.38 (s), 1.40 (s), 1.43 (s).

2,2',2''-(10-(7-Carboxyheptyl)-1,4,7,10-tetraazacyclododecane-1,4,7-triyl)triacetic acid (8): Compound **S2** (0.17 mmol, 123 mg) was dissolved in DCM (2 mL). Tri-*iso*-propyl silane (1.0 mmol, 0.21 mL) and HCl in Et_2O (2 m, 7 mL) were added to the solution. The reaction mixture was stirred at room temperature overnight. The volatiles were evaporated under reduced pressure and Et_2O added to the residue. The product was filtered off and re-dissolved in MeOH. The solution was concentrated under reduced pressure and the residue triturated with Et_2O. The product was filtered off again to afford a white solid (88 mg, 97% calculated as mono hydrochloride salt). ^{1}H NMR (400 MHz, D_2O): δ 1.37 (s, 6H), 1.61 (quint, 2H), 1.76 (br s, 2H), 2.4 (t, 2H), 3.02-3.18 (overlapping m, 6H), 3.27 (t, 2H), 3.35-3.44 (overlapping m, 4H), 3.48-3.54 (overlapping m, 6H), 3.61-3.76 (overlapping m, 4H), 4.05 (s, 2H). ^{13}C NMR (100 MHz, D_2O): δ 22.7, 24.0, 25.4, 27.7, 27.7, 27.7, 27.8, 33.5, 33.6, 48.0, 48.3, 49.8, 51.6, 52.0, 52.9, 54.6, 65.4, 169.2, 174.2, 177.7, 179.2.

2. Quantum yield determination

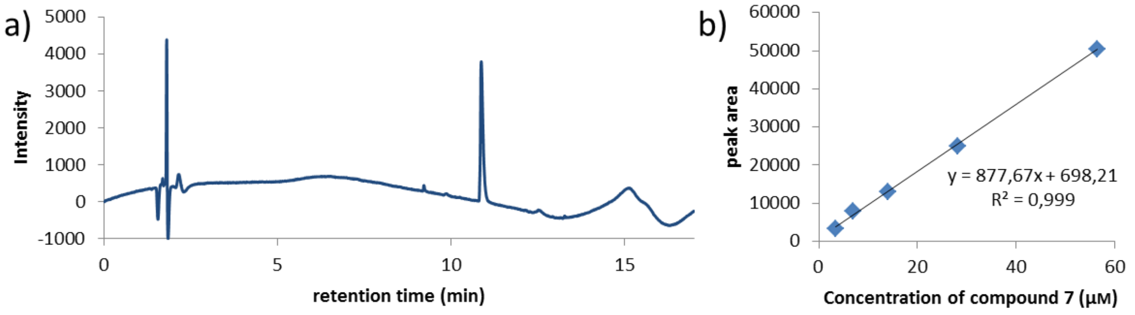

Figure S1. Quantification of compound **7** by UPLC. a) chromatogram recorded at λ = 360 nm for the solution of compound **7** (0.565 mM in acetonitrile), after 1:15 dilution with MilliQ water. The area of the peak with a retention time of 10.8 min was used for quantification. b) Calibration curve for the quantification of compound **7** by UPLC.

3. NMRD profiles

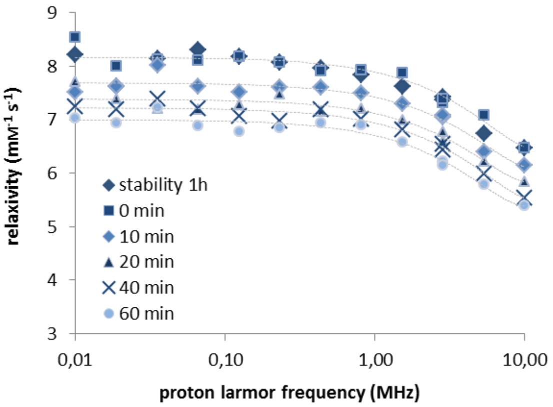

Figure S2. NMRD profiles of a sample containing **Gd-1** before irradiation (0 min), after 1 h in the dark (stability 1 h) and after irradiation for indicated time points (10; 20; 40; 60 min). See supporting tables 1 and 2 for numerical data.

4. Relaxometry data at different magnetic field strength

Table S1. Molar relaxivity (s^{-1} mM^{-1}) profiles of a sample of **Gd-1** in TBS buffer pH 7.5 before irradiation (0 min), after 1 h in the dark (stability 1 h) and after irradiation for indicated time points (10; 20; 40; 60 min). The standard deviation represents the uncertainty of fitting the T1 curve to the experimental data.

Larmor frequency (MHz)	**0.010**	**0.019**	**0.035**	**0.066**	**0.123**	**0.231**	**0.433**	**0.811**	**1.52**	**2.85**	**5.34**	**10.0**
0 min (dark)	8.54 ±0.28	8.00 ±0.20	8.13 ±0.31	8.10 ±0.30	8.18 ±0.04	8.07 ±0.05	7.91 ±0.05	7.92 ±0.05	7.87 ±0.09	7.31 ±0.11	7.08 ±0.06	6.47 ±0.04
0 min (dark 1 h)	8.22 ±0.21	7.63 ±0.30	8.14 ±0.25	8.30 ±0.20	8.19 ±0.05	8.07 ±0.06	7.96 ±0.06	7.84 ±0.06	7.63 ±0.06	7.42 ±0.07	6.74 ±0.08	6.47 ±0.04
10 min	7.51 ±0.19	7.61 ±0.16	8.03 ±0.22	7.63 ±0.18	7.51 ±0.07	7.60 ±0.04	7.61 ±0.03	7.50 ±0.05	7.30 ±0.06	7.04 ±0.07	6.41 ±0.04	6.15 ±0.02
20 min	7.72 ±0.31	7.39 ±0.13	7.22 ±0.19	7.19 ±0.23	7.29 ±0.04	7.48 ±0.05	7.16 ±0.06	7.20 ±0.05	6.99 ±0.09	6.78 ±0.08	6.22 ±0.06	5.84 ±0.04
40 min	7.24 ±0.30	7.20 ±0.12	7.39 ±0.24	7.21 ±0.16	7.06 ±0.05	6.98 ±0.04	7.19 ±0.04	7.02 ±0.05	6.82 ±0.04	6.54 ±0.07	5.99 ±0.04	5.54 ±0.04
60 min	7.03 ±0.28	6.94 ±0.14	7.23 ±0.24	6.89 ±0.20	6.78 ±0.05	6.85 ±0.04	6.94 ±0.07	6.90 ±0.06	6.57 ±0.07	6.22 ±0.09	5.79 ±0.05	5.39 ±0.04

Table S2. Molar relaxivity (s^{-1} mM^{-1}) profile of a sample of **Gd-8** in TBS buffer pH 7.5. The standard deviation represents the uncertainty of fitting the T1 curve to the experimental data.

Larmor frequency (MHz)	**0.010**	**0.021**	**0.046**	**0.100**	**0.215**	**0.464**	**1.00**	**2.15**	**4.64**	**10.0**
Gd-8	6.58 ±0.29	6.72 ±0.15	6.61 ±0.17	6.43 ±0.06	6.59 ±0.05	6.33 ±0.04	6.39 ±0.05	6.26 ±0.05	5.90 ±0.05	5.02 ±0.02

Table S3. Molar relaxivity (s^{-1} mM^{-1}) and standard deviation (SD) of a sample of **Gd-1** in TBS buffer pH 7.5 in response to irradiation for the indicated time points (0; 3; 60 min), at 1.5T (63.87 MHz) and 3.0T (127.74 MHz).

Magnetic field strength	**1.5T**	**1.5T**	**3.0T**	**3.0T**
	**MEAN**	**SD**	**MEAN**	**SD**
**0 min**	8.58	0.19	8.50	0.10
**3 min**	7.79	0.16	7.73	0.11
**60 min**	6.93	0.10	6.85	0.06

5. Determination of free gadolinium(III) concentration

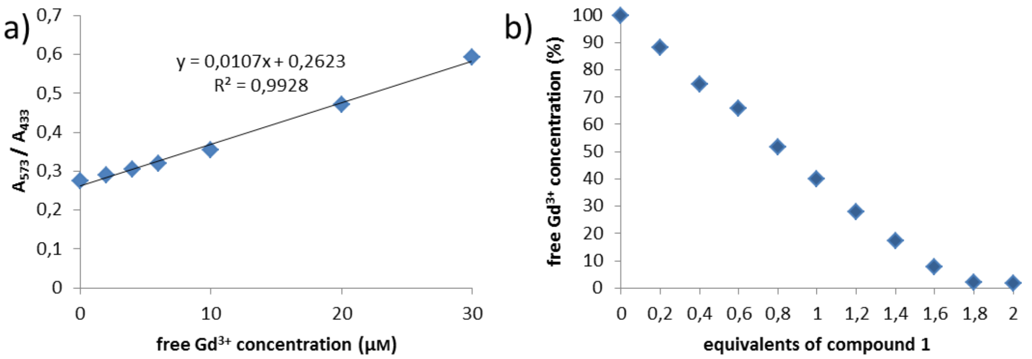

Figure S3. Quantification of free Gd^{III}. a) Calibration curve showing the ratio of absorbance intensity at λ = 573 nm and λ = 433 nm for increasing Gd^{III} concentration in the presence of xylenol orange (0.60 mM). b) Percentage of free Gd^{III} in the presence of increasing equivalents of compound **1**.

6. NMR spectra

1-(4,5-Dimethoxy-2-nitrophenyl)-2-oxo-2-((2-oxo-2-(prop-2-yn-1-ylamino)ethyl)amino)-ethyl 8-bromooctanoate (3):

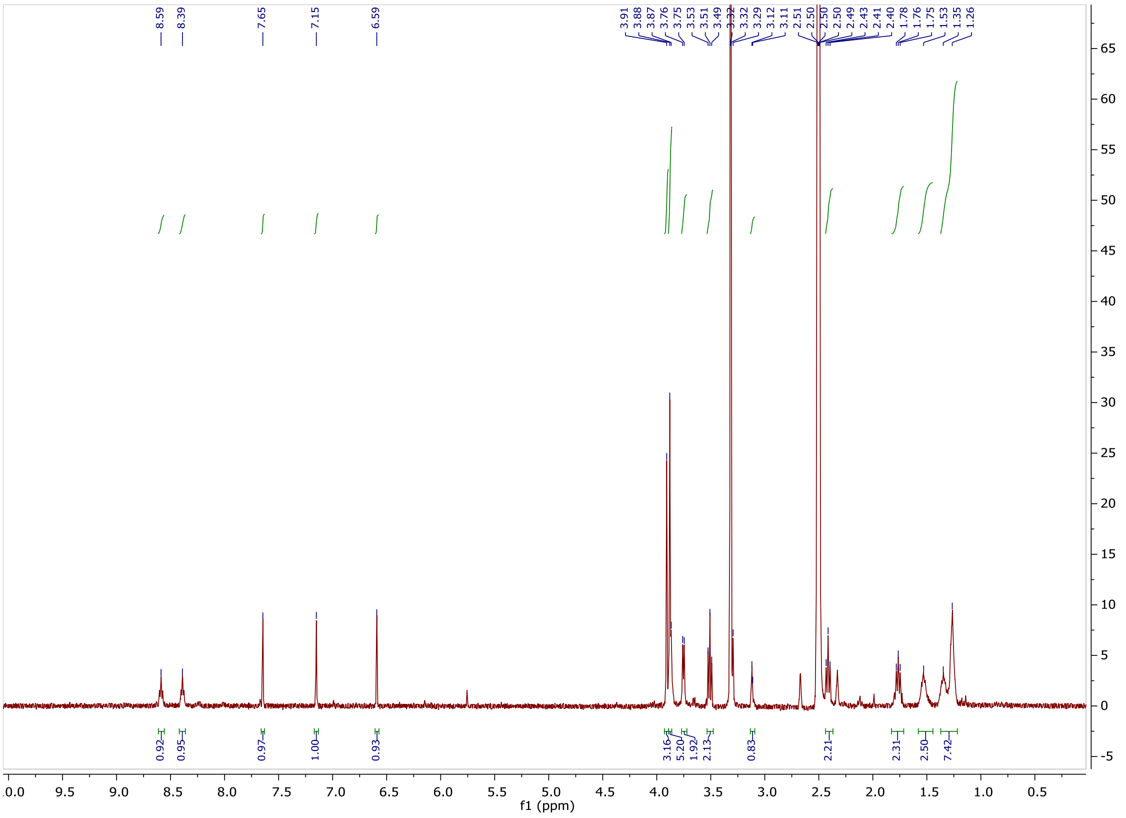

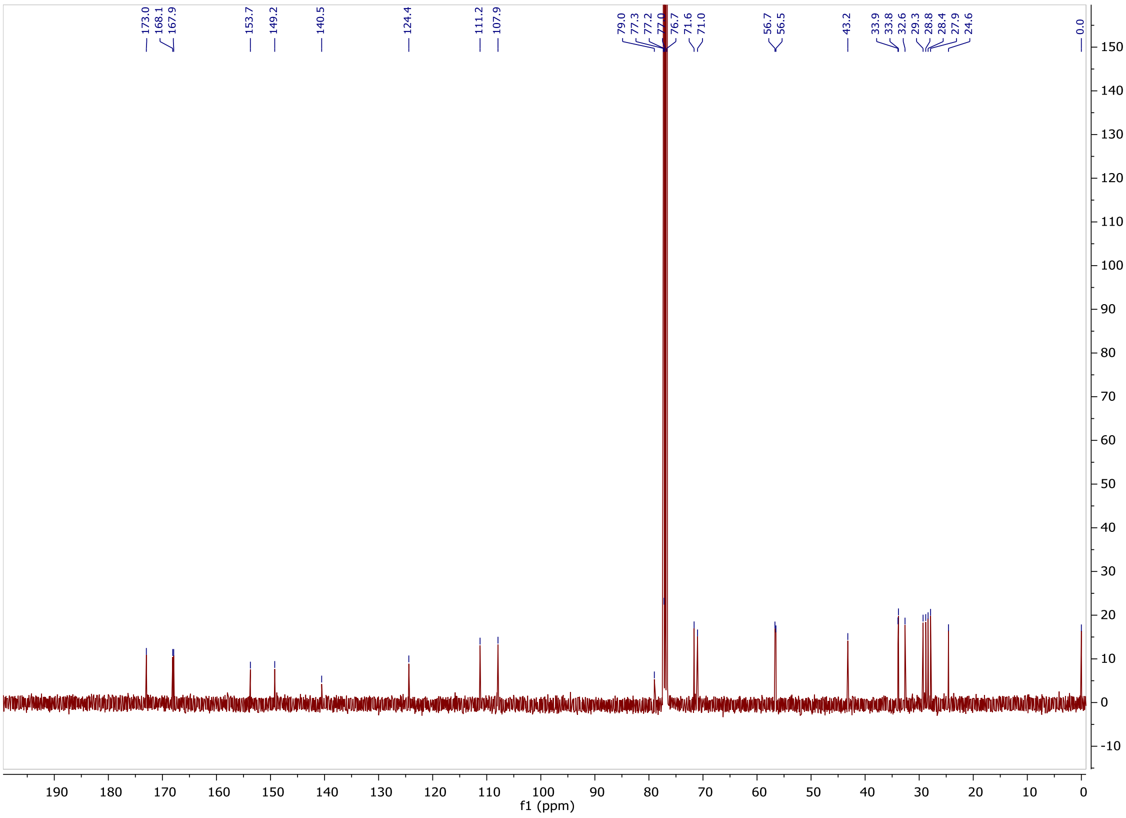

1-(4,5-Dimethoxy-2-nitrophenyl)-2-((2-(((1-(2-(2-(2-methoxyethoxy)ethoxy)ethyl)-1*H*-1,2,3-triazol-4-yl)methyl)amino)-2-oxoethyl)amino)-2-oxoethyl 8-bromooctanoate (5):

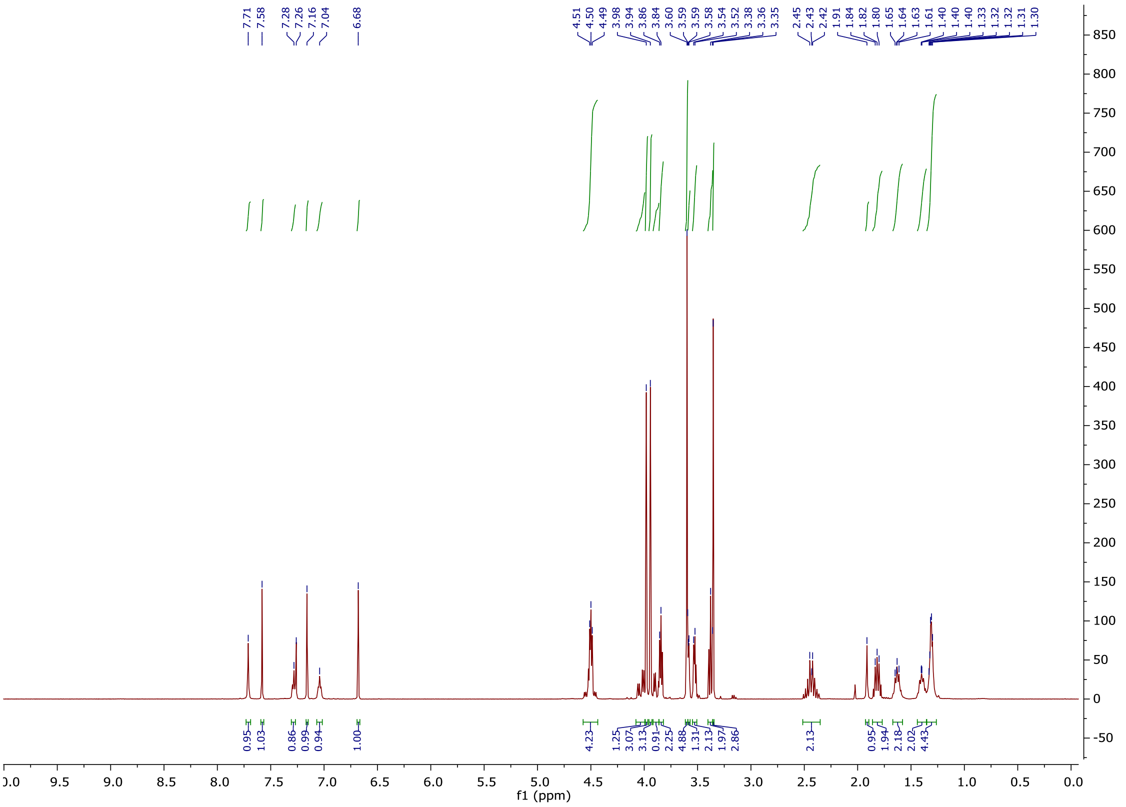

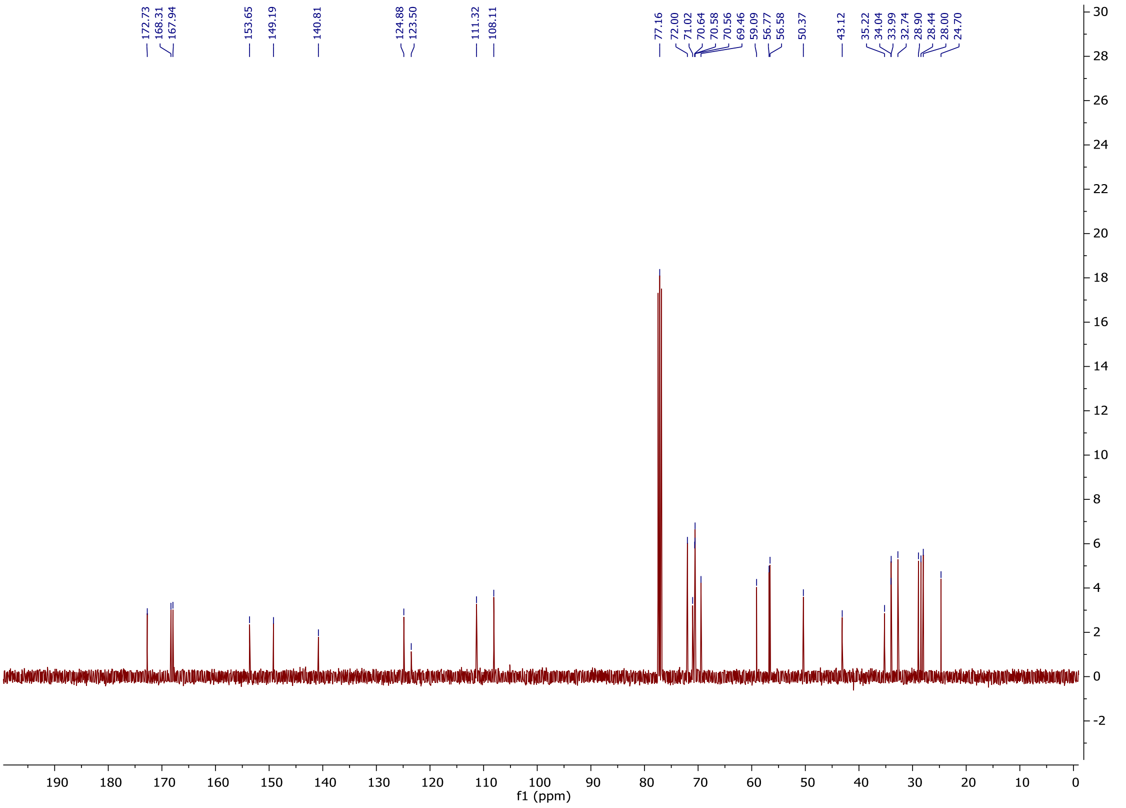

Tri-*tert*-butyl 2,2',2''-(10-(8-(1-(4,5-dimethoxy-2-nitrophenyl)-2-((2-(((1-(2-(2-(2-methoxy ethoxy)ethoxy)ethyl)-1*H*-1,2,3-triazol-4-yl)methyl)amino)-2-oxoethyl)amino)-2-oxoethoxy)-8-oxo octyl)-1,4,7,10-tetraazacyclododecane-1,4,7-triyl)triacetate (7):

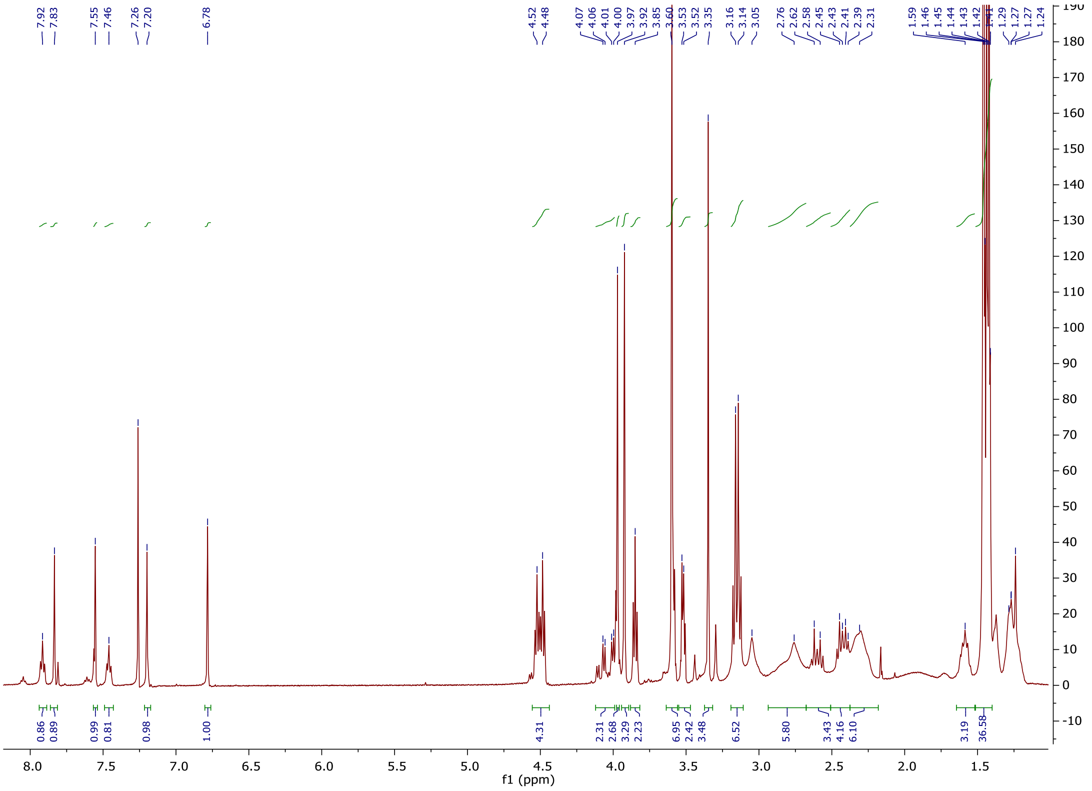

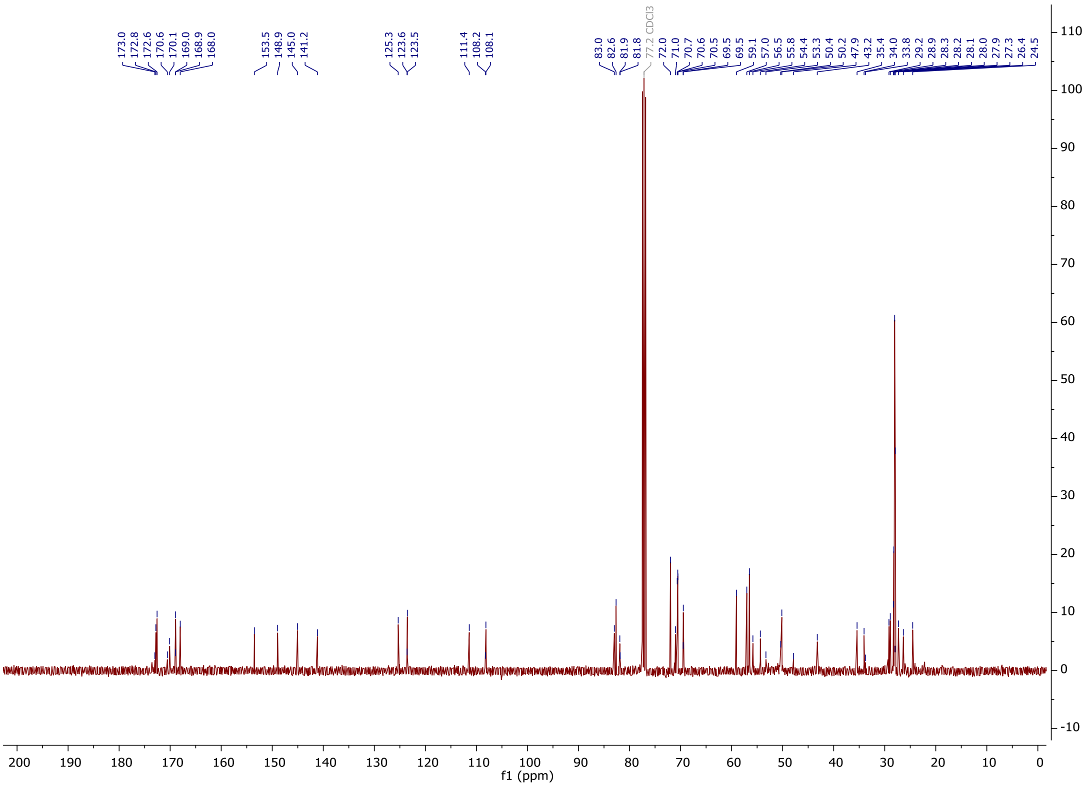

2,2',2''-(10-(8-(1-(4,5-Dimethoxy-2-nitrophenyl)-2-((2-(((1-(2-(2-(2-methoxy-ethoxy)ethoxy) ethyl)-1*H*-1,2,3-triazol-4-yl)methyl)amino)-2-oxoethyl)amino)-2-oxo-ethoxy)-8-oxooctyl)-1,4,7,10-tetraazacyclododecane-1,4,7-triyl)triacetic acid (1):

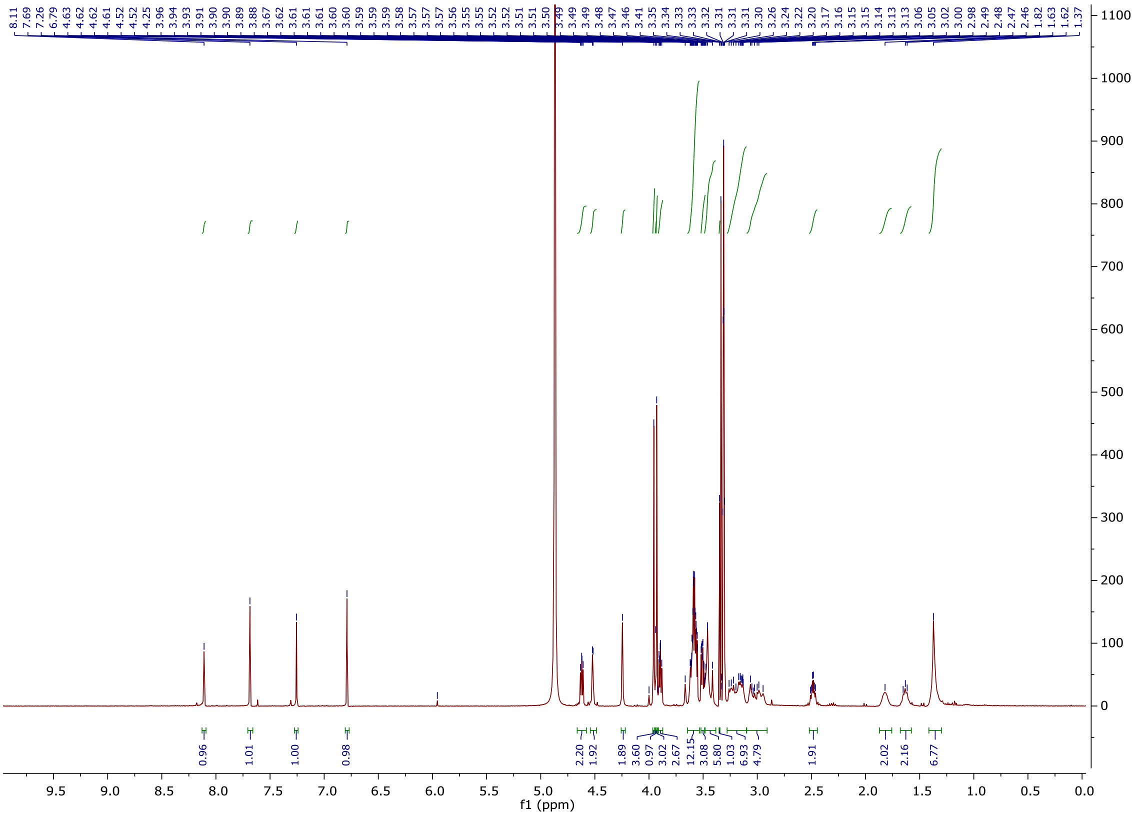

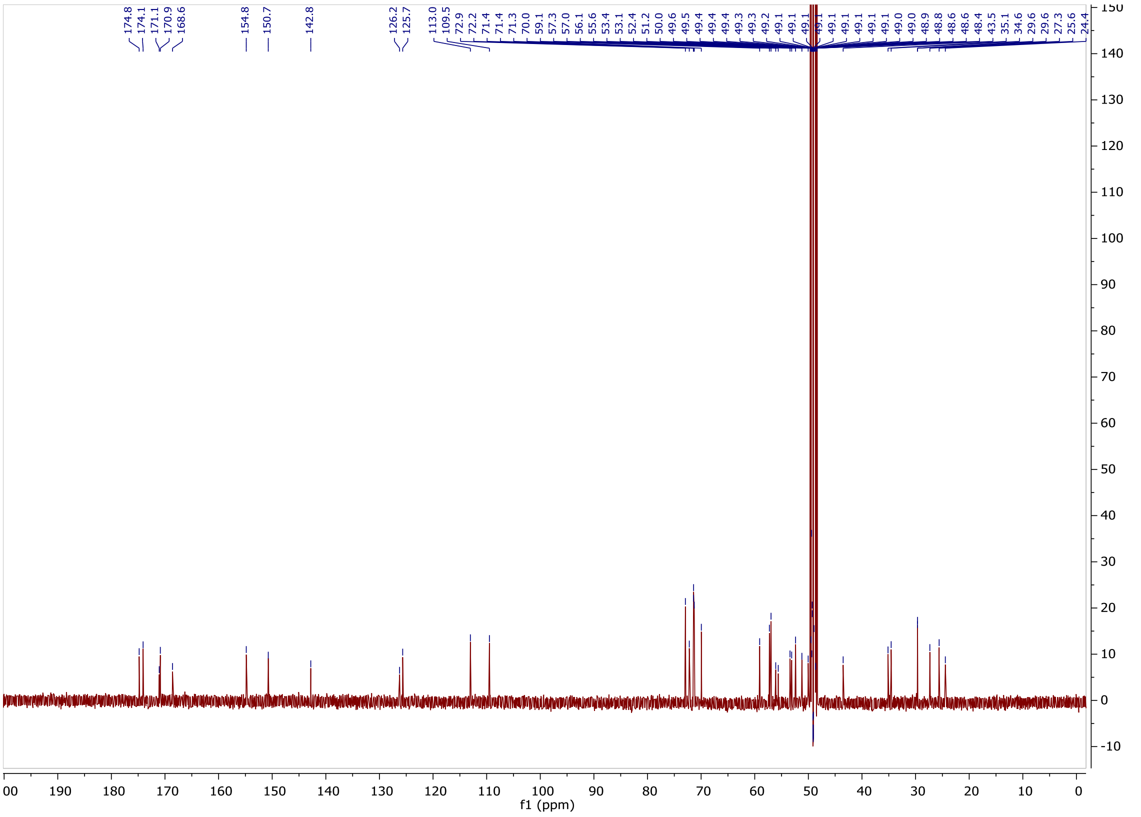

References:

1. Dömling, A.; Beck, B.; Fuchs, T.; Yazbak, A. Parallel synthesis of arrays of amino-acid-derived isocyanoamides useful as starting materials in IMCR. *J. Comb. Chem.* **2006**, *8*, 872–880.

2. Wang, Y.; Patil, P.; Dömling, A. Easy Synthesis of Two Positional Isomeric Tetrazole Libraries. *Synthesis* **2016**, *48*, 3701–3712.

3. Borissov, A.; Marques, I.; Lim, J.Y.C.; Félix, V.; Smith, M.D.; Beer, P.D. Anion Recognition in Water by Charge-Neutral Halogen and Chalcogen Bonding Foldamer Receptors. *J. Am. Chem. Soc.* **2019**, *141*, 4119–4129.

4. Jagadish, B.; Brickert-Albrecht, G.L.; Nichol, G.S.; Mash, E.A.; Raghunand, N. On the synthesis of 1,4,7-tris(tert-butoxycarbonylmethyl)-1,4,7,10- tetraazacyclododecane. *Tetrahedron Lett.* **2011**, *52*, 2058–2061.

5. Jacquot, C.; McGinley, C.M.; Plata, E.; Holman, T.R.; van der Donk, W.A. Synthesis of 11-thialinoleic acid and 14-thialinoleic acid, inhibitors of soybean and human lipoxygenases. *Org. Biomol. Chem.* **2008**, *6*, 4242–4252.
